# A method to map the interaction network of the nuclear lamina with genetically encoded photo-crosslinkers *in vivo*

**DOI:** 10.3389/fchem.2022.905794

**Published:** 2022-08-30

**Authors:** Petra Neumann-Staubitz, Daniel Kitsberg, Amnon Buxboim, Heinz Neumann

**Affiliations:** ^1^ University of Applied Sciences Darmstadt, Darmstadt, Germany; ^2^ Institute of Life Science, Hebrew University of Jerusalem, Jerusalem, Israel; ^3^ Rachel and Selim Benin School of Computer Science and Engineering, Hebrew University of Jerusalem, Jerusalem, Israel; ^4^ Alexander Grass Center for Bioengineering, Hebrew University of Jerusalem, Jerusalem, Israel

**Keywords:** genetic code expansion, unnatural amino acids, photo-crosslinking, nuclear lamina, laminA/C, laminB1

## Abstract

Lamins are intermediate filaments that assemble in a meshwork at the inner nuclear periphery of metazoan cells. The nuclear periphery fulfils important functions by providing stability to the nuclear membrane, connecting the cytoskeleton with chromatin, and participating in signal transduction. Mutations in lamins interfere with these functions and cause severe, phenotypically diverse diseases collectively referred to as laminopathies. The molecular consequences of these mutations are largely unclear but likely include alterations in lamin-protein and lamin-chromatin interactions. These interactions are challenging to study biochemically mainly because the lamina is resistant to high salt and detergent concentrations and co-immunoprecipitation are susceptible to artefacts. Here, we used genetic code expansion to install photo-activated crosslinkers to capture direct lamin-protein interactions *in vivo*. Mapping the Ig-fold of laminC for interactions, we identified laminC-crosslink products with laminB1, LAP2, and TRIM28. We observed significant changes in the crosslink intensities between laminC mutants mimicking different phosphorylation states. Similarly, we found variations in laminC crosslink product intensities comparing asynchronous cells and cells synchronized in prophase. This method can be extended to other laminC domains or other lamins to reveal changes in their interactome as a result of mutations or cell cycle stages.

## Introduction

The nuclear lamina is a dense three-dimensional meshwork that lines the inner nuclear membrane of metazoa. The lamina not only provides mechanic stability to the nucleus as a structural element, it also organizes the chromatin by anchoring it, interconnects the cytoskeleton with inner nuclear proteins and serves as an important signaling hub (Xie and Burke, 2016; Leeuw et al., 2018).

Lamins, the major components of the nuclear lamina (Gruenbaum and Foisner, 2015; Xie and Burke, 2016; Leeuw et al., 2018), are distinguished by their biochemical properties in A- and B-types. The expression of different lamin types correlates with the complexity of the organism, for example, laminC is only found in mammals (Gruenbaum and Foisner, 2015). Encoded by the same gene, laminC is a shortened splice variant of laminA, which is heavily post-translationally modified. Although a large set of proteins are found in association with both A-type lamin isoforms, some differ significantly, suggesting partially non-overlapping functions (Xie et al., 2016).

Lamins show a tripartite domain structure composed of a short N-terminal head-domain, a long α-helical rod-domain and a long C-terminal domain including the Ig-fold domain (Gruenbaum and Foisner, 2015; Xie and Burke, 2016; Leeuw et al., 2018). The Ig-fold of human laminA/C is 116 amino acids long (aa 436–552) and composed of β-strands forming a sandwich structure (Dhe-Paganon et al., 2002). In a cryo-electron tomographic image, the Ig-fold forms a visually distinct structure (Turgay et al., 2017). Its position within the lamina makes it a prime candidate for an interaction hot spot with other proteins.

Over the years, a number of diseases, collectively named laminopathies, have been identified that are caused by mutations predominantly in the laminA/C gene (Gruenbaum and Foisner, 2015; Leeuw et al., 2018). Consequently, there has been a growing interest in understanding the organization of the lamin network, the processes within the lamina, and their role within the cell.

A well-investigated process is the release of lamins from the lamina by multiple phosphorylation. This process is responsible for lamina breakdown during mitosis and is a key modification in interphase in response to mechanical stress (Machowska et al., 2015; Torvaldson et al., 2015; Athirasala et al., 2017). Lamina plasticity is modulated by changing its thickness, which is controlled by local mobilization or incorporation of A-type lamins. As a result of phosphorylation, the nucleoplasmic fraction of A-type lamins is increases (Athirasala et al., 2017).

Structural analysis of the nuclear lamina by cryo-electron microscopy, NMR and crystallography has provided a good understanding of its three-dimensional organization (Pecorari et al., 2017; Turgay and Medalia, 2017; Xie and Burke, 2017). Biochemically, the lamina is an extremely stable filamentous meshwork resistant to common extraction procedures, which hinders the identification of lamin-associated proteins by conventional pull-down and co-immunoprecipitation (Roux et al., 2012; Xie and Burke, 2016; Bar et al., 2018). At present, two methods with different scopes of application are available for *in vivo* studies: Biotinylation by antibody recognition (BAR) (Bar et al., 2018) and proximity-dependent biotinylation of proteins by the promiscuous biotin ligase BirA (BioID) (Roux et al., 2012; Mehus et al., 2016; Xie et al., 2016). Whereas BAR is applied in fixed cells and primary tissues, BioID requires cloning of a BirA-fusion protein. Combined with mass spectrometry, these methods have delivered a promising set of lamin-interacting factors.

Unfortunately, such methods are limited in their spatial resolution, provide largely qualitative results and cannot distinguish between physical interactions and proximity. To fill this gap, we set out to establish the incorporation of photo-activatable crosslinker amino acids in lamin proteins of mammalian cells by genetic code expansion (Neumann-Staubitz and Neumann, 2016; Coin, 2018). These amino acids facilitate covalently trapping protein-protein interactions in living cells (Wilkins et al., 2014; Hoffmann et al., 2018). Their incorporation is achieved by the heterologous expression of an orthogonal aminoacyl-tRNA synthetase (AARS)/tRNA_CUA_ pair in the host system. The AARS is designed to acylate the tRNA with the crosslinker amino acid, while the tRNA is engineered to suppress amber stop codons inserted at the desired site into the mRNA of a protein of choice.

In this study, we used the orthogonal *Ec*BPARS/tRNA_CUA_ system derived from *E. coli* TyrRS (Seidel and Coin, 2018) to install the photo-activatable crosslinker amino acid p-benzoyl-phenylalanine (pBPA) in laminC. After light activation, pBPA crosslinks proteins which are in very close contact [less than 1 nm (Sato et al., 2011)]. We identified interactions of the Ig-domain of laminC with laminA/C, laminB, TRIM28 and LAP2 and introduced phosphomimetic laminC mutations to quantify their impact on crosslinking efficiencies. This work provides a basis to investigate lamin-protein interactions in living cells with respect to cell cycle stages, disease-related mutations and environmental conditions.

## Materials and methods

### Preparing laminC constructs

laminC was amplified from mouse cDNA by PCR and subsequently cloned into the pEGFP-N1 vector *via* NheI/SpeI and BamHI/BglII to obtain a C-terminal GFP fusion. For Western blot analysis, the eGFP in the pEGFP-N1 vector was replaced by a 3xFlag-tag, amplified by PCR, *via* BamHI/NotI sites. Analogous to the eGFP-tagged laminC constructs, a C-terminal 3xFlag-tagged laminC fusion was generated. The QuikChange method was used to replace a codon of choice by an amber stop codon. The amber suppressor plasmid containing four copies of *B. stearothermophilus* Tyr-tRNA_CUA_ and the corresponding pBPA-specific AARS (pIRE4-4xU6-BstYam-PGKcoBPARS) (Seidel and Coin, 2018) was a gift from Irene Coin.

### Cell culture

HEK293 cells were maintained in 5% CO_2_, 37°C in DMEM+GlutaMAX^™^-I medium (Gibco Life Technologies, by Thermo Fisher Scientific, 10569–010, containing 4.5 g/L glucose, 110 mg/L pyruvate) supplemented with 10% heat-inactivated fetal calf serum, 1000 units/ml penicillin and 1 mg/ml streptomycin.

### Transient transfection

24 h prior to transfection, 4 ml of 1 × 10^5^ HEK293 cells were seeded in 6 cm Ø polylysine-coated (Greiner-Bio-one, Cellstar; Poly-L-Lysine Merck L7240, 1:100 diluted) petri dishes resulting in 40–50% confluent grown cells. A modified polyethylenimine method [PEI; original protocol in (Boussif et al., 1995)] was used: The medium was replaced by 5 ml fresh medium containing 1 mM pBPA (para-benzoyl-phenylalanine, Bachem F-2800). For each dish, a transfection mix in a small sterile tube was prepared: First 635 µl DMEM+GlutaMAX^™^-I medium w/o serum, then 6.25 µg of each plasmid DNA was added, mixed, then 12.5 µl of 2 mg/ml neutralized PEI was included, mixed thoroughly and incubated 5 min at RT. Finally, the content of one tube was applied onto one dish dropwise and equally. Cells were incubated for 24 h at 37°C and at 5% CO_2_.

### Microscopy

Cells were grown on poly-lysine-treated coverslips in 6-well plates. Each laminC-GFP amber mutant plasmid was cotransfected with the amber suppressor plasmid as transient transfection by the PEI-method. For the laminC-GFP amber mutant screen two samples were prepared with or without addition of 1 mM pBPA. 24 h post transfection (p.t.) the medium was replaced by fresh medium with or without pBPA. For the lmnC-T534-3xFlag cdk1 mutants, only one dish with medium with 1 mM pBPA was required. For the GFP-constructs, cells were washed two times with PBS, fixed for 15 min at RT with 4% paraformaldehyde and permeabilized in 0.1% Triton X-100 in PBS for 15 min at RT. Cells were incubated for 15 min with PBS containing 1 μg/ml DAPI. The different lmnC-T534-3xFlag constructs were immune-stained by fixing the cells with 4% paraformaldehyde, blocking with blocking buffer (3% BSA, 0.2% Triton X-100 in PBS), incubation with the primary antibody against the Flag-tag (1:1000 rabbit, F7425 Sigma-Aldrich) in blocking buffer for 2 h at RT, then washing 3 times for 5 min in PBS followed by incubation with a secondary Cy3-conjugated anti-rabbit antibody (1:1000 Jackson Immuno Research, 111-165-144) and 1 μg/ml DAPI for 1 h at RT. Thereafter, the coverslips were dried, mounted and analyzed by LSM (Zeiss LSM800/510, 40x or ×63 oil objective).

### Crosslinking and sample preparation

For comparison, two dishes per sample are required and prepared as specified under “transient transfection”. 24 h p.t., the medium was replaced by fresh medium containing 1 mM pBPA. To prepare samples with or without crosslinking, the medium was removed, the petri dishes were washed once with PBS (phosphate buffered saline pH 7.4), covered with 2 ml PBS and kept on ice. One dish was transferred to a full ice box, the lid was removed and the cells were exposed 15 min to UV-light (VILBER Lourmat VL-208.BL, with 2 × 8 W 365 nm tubes, distance between the dish and the UV-lamp was approximately 3 cm). Subsequently, PBS was aspirated from all dishes, 250 µl lysis buffer (50 mM TrisBase pH 8, 150 mM NaCl, 0.1% NP40, 1 μl/ml Pierce^TM^ Universal Nuclease) was added and cells were allowed to lyse on ice for 10 min. Cell lysates were collected by scraping the dish with a cell scraper, SDS was added to a final concentration of 1% and the lysate was incubated 5 min at RT and 300 rpm agitating. The soluble supernatant was collected after centrifugation at 15.000 rpm for 5 min at RT. Samples were mixed with 5x SDS-crack buffer (0.313 M Tris/HCl pH 6.8, 10% SDS, 50% glycerol, 0.05% Bromophenol blue, and 0.25 mM DTT), heated at 95°C for 12 min and used for Western blot analysis or stored at −20°C.

### SDS-PAGE and Western blotting

Unless otherwise noted, to detect crosslinked proteins, 3%–8% Tris-Acetate gradient gels (NuPAGE^TM^/Invitrogen/Thermo Fisher Scientific) run in 1x Tris Acetate NuPAGE™ running buffer were used followed by blotting with NuPAGE™ transfer buffer (Thermo Fisher Scientific) supplemented with 20% methanol. For proteins smaller than 70 kDa, 10% Laemmli gels and Towbin transfer buffer containing 20% methanol was applied. Blotting onto PVDF membranes (Immobilon-P, pore size 0.45 µm, Merck IPVH00010) was carried out for 2.5 h, 280 mA at 4°C. Subsequently, the membrane was blocked in 5% skimmed milk in PBS followed by an incubation with the primary antibody (4°C, ON), washing and then incubation with secondary HRP-coupled antibody (1 h, RT). For anti-β-actin and anti-cyclinB1 detection, luminol solution “Prime”, and for all other detections, luminol solution “Select” both from Amersham/GE Healthcare (ECL Prime/Select Western Blotting Detection Reagent) were used. All antibodies were used in 5% skimmed milk in PBS; primary antibodies: anti-β-actin (1:5000 mouse, clone AC-15, A5441 Sigma-Aldrich), anti-cyclinB1 (1:1000 mouse, clone V152, 647901 BioLegend), anti-Flag (1:10000 rabbit, F7425 Sigma-Aldrich), anti-laminB1 (1:5000, rabbit, ab194109 Abcam), anti-LAP2 (1:1000, Merck 06–1002) anti-KAP1 (TRIM28; 1:1000, rabbit, 15202-1-AP Proteintech Europe); secondary antibodies: anti-mouse-HRP (1:10000, SIGMA A4416), anti-rabbit-HRP (1:10 000, Abcam 6721).

### Test of pBPA dependent incorporation

For transient transfection, the plasmid encoding BPARS and four copies of the corresponding tRNA_CUA_ was cotransfected with a laminC amber mutant plasmid. Two dishes per experiment were prepared. Cells in one dish were grown in medium without pBPA, the other received medium containing 1 mM pBPA. 24 h p.t. the medium was replaced by fresh medium with or without 1 mM pBPA. Cells were not crosslinked but the protocol for sample preparation was used and 1x RIPA buffer served as lysis buffer. A 4%–12% Bis-Tris gel (NuPAGE^TM^/Invitrogen/Thermo Fisher Scientific) run in 1x MOPS NuPAGE™ running buffer was used for protein separation. Flag-tagged pBPA-containing laminC proteins were detected by the Western blot method.

### UV-dependent crosslinking of cdk1-mutants

For all experiments, the plasmid encoding BPARS and four copies of the corresponding tRNA_CUA_ was cotransfected with either a plasmid containing a laminC-3xFlag amber mutant or with a plasmid containing mutated cdk1 sites (S22 and S392 both either genetically converted to alanine or to aspartate) along with an amber mutation. Cells were incubated for 24 h in 1 mM pBPA medium and then crosslinked as described above. Here, all samples were lysed in PBS with 0.2% Triton-X100 and analyzed by SDS-PAGE and Western blot.

### Synchronizing cells

To prepare cells for synchronization and later early M-phase block, the transient transfection protocol was followed. In petri dishes with cells not used for synchronization (asynchronized control), the medium was replaced 24 h p.t. for another 24 h and the protocol for crosslinking and sample preparation was carried out. To synchronize with SLTC (S-Trityl-L-cysteine, T7232 LKT Laboratories, 50 mM stock in DMSO), the medium was replaced 24 h p.t. by new medium containing 100 µM SLTC and 1 mM pBPA and incubated for 20 h at 37°C and 5% CO_2_. Then samples were processed for crosslinking and WB using 0.2% Triton-X100 as lysis buffer. For blocking with nocodazole, a thymidine-block (100 µM final concentration, T1895 Sigma-Aldrich) was applied for 21 h in medium containing 1 mM pBPA; the block was released for 3 h in 1 mM pBPA containing medium. For the M-phase block, 1 mM pBPA containing medium was supplemented with 10 μg/ml nocodazole (10 mg/ml stock in DMSO, M1404 Sigma-Aldrich) was given to the cells for 20 h. Then, sample processing was completed following the crosslinking and sample preparation protocol for further SDS-PAGE and Western blot analyses.

## Results

### Screen for laminC amber mutants

In order to investigate the dynamic network of the nuclear lamina, a method is required that will freeze interactions of the lamins in action and in their natural environment. Therefore, we set out to establish genetic code expansion for lamins in mammalian cells to introduce photo-activated crosslinker amino acids at permissive positions in the lamin proteins. We initially chose laminC for the incorporation of the crosslinker amino acids as it is not farnesylated like the other lamins.

**FIGURE 1:**
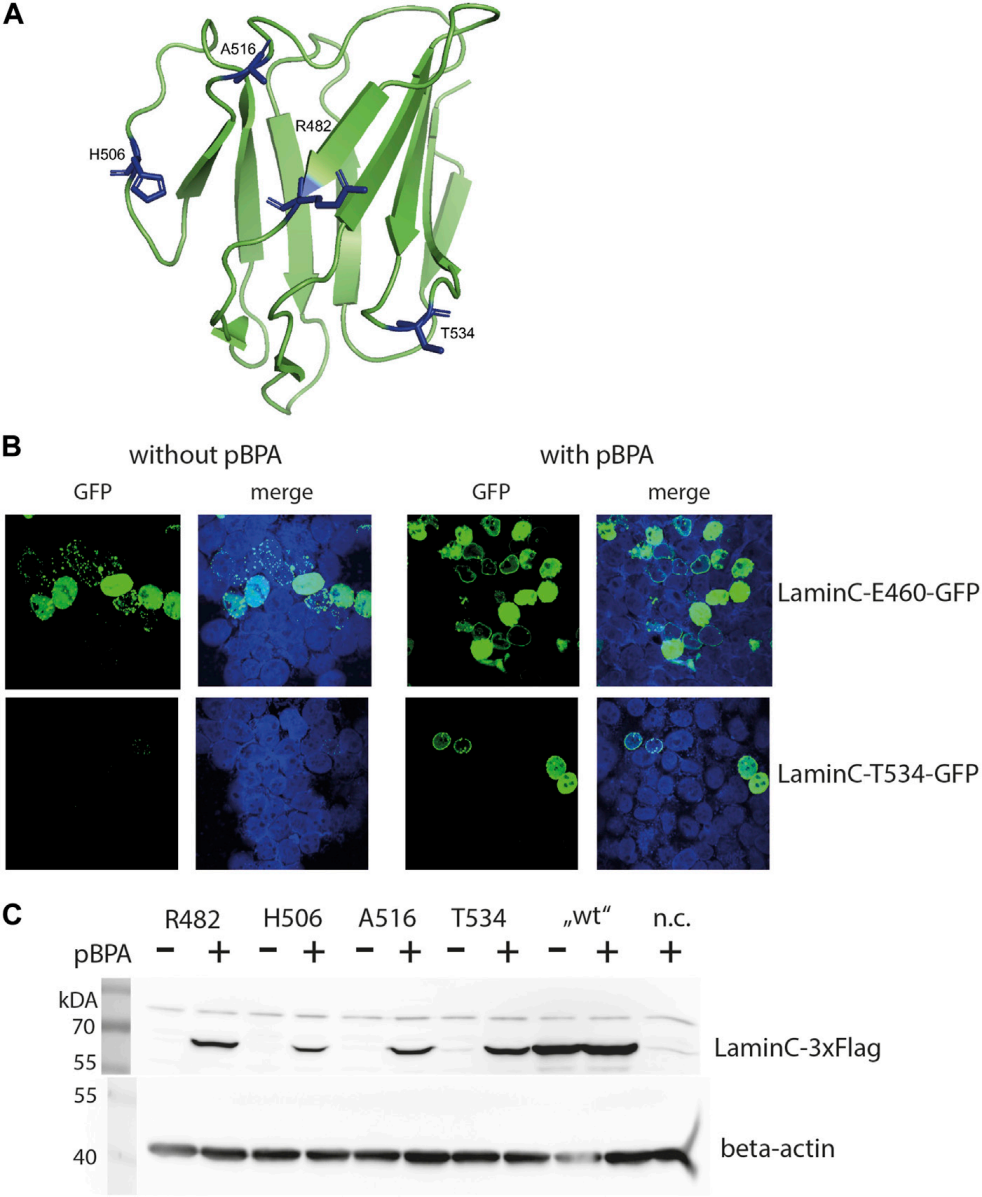
pBPA dependent incorporation. **(A)** Structure of the laminA/C-Ig fold with residues used for pBPA incorporation indicated in blue (Image created with PyMol 2.2.0 and pdb-file 1IFR). **(B)** HEK293 cells cotransfected with a pLmnC-GFP construct with an amber mutation at the indicated site and the amber suppressor plasmid, incubated w/wo pBPA, and analyzed by LSM. laminC-GFP fusion proteins are shown in green, DNA is colored in blue. **(C)** HEK293 cells were transiently transfected wifigureth the amber suppressor plasmid and a pLmnC-3xFlag construct containing an amber codon as indicated. The cells were grown in the presence or absence of pBPA and prepared for SDS-PAGE and Western blot analysis. Lysate of non-transfected HEK293 cells served as negative control (n.c.). The upper panel shows a Western blot for anti-Flag and the lower panel for the loading control anti-β-actin.

Within the laminA/C molecule, it is believed that the Ig-fold in the C-terminal tail is a hot spot for interactions. Based on the structure of the Ig-domain of laminA/C, (DOI:10.2210 / pdb1FR / pdb (Dhe-Paganon et al., 2002)) we selected 11 surface-exposed residues distributed across both sides of the β-sheet sandwich (Figure 1A) for an amber mutant screen. First, we tested the impact of substitution of the selected residues with pBPA on laminC localization and expression pattern. Therefore, laminC was C-terminally tagged with GFP and constitutively expressed under control of a CMV promoter. This plasmid was cotransfected with the amber suppressor plasmid for pBPA incorporation.

All C-terminal GFP fused laminC proteins localized to the nucleus in transiently transfected HEK293 cells grown in the presence of pBPA (Figure 1B and Supplementary Figure S1). Correct localization to the lamina was observed when residues R470, R482, T488, H506, A516 or T534 were replaced by pBPA. laminC mutants containing pBPA in place of S437, K450 or V494, however, produced a punctate pattern in the periphery of the nucleus, and the mutants K450, N459, and E460 showed a wide range of expression levels from hardly detectable to strongly overexpressed.

We also tested the dependence of laminC expression on the availability of pBPA in our microscopic screen, although incorrect incorporation of natural amino acids, e.g., tryptophan, in response to the amber codon would not necessarily hinder our search for crosslink products. In this respect, amber mutants N459 and E460 showed strong pBPA-independent GFP fluorescence, whereas the pBPA-independent signal was moderate in T488 and A516 and undetectable in S437, K450, R470, V494, and T534.

Based on these observations, we selected four amber mutants (R482, H506, A516, and T534) showing correct localization to the lamina and pBPA-dependent expression for further analyses. Figure 1B gives examples of desired (laminC-T534-GFP) and undesirable (lmnC-E460-GFP) expression patterns. The selected positions locate in the loop regions and on both sides of the Ig-fold and should therefore represent different interaction surfaces of this domain (Figure 1A). All of these mutants showed pBPA-dependent expression of laminC in Western blot using 3xFlag-tagged constructs (Figure 1C).

This differs from the microscopic analysis where all selected mutants except T534 showed slight or moderate pBPA-independent incorporation (Supplementary Figure S1). This may be a result of GFP being a stable protein, which may contribute to a slower turnover rate of GFP-fused proteins. Expression levels of laminC-3xFlag R482BPA and T534BPA are approximately 45%, H506BPA 10% and A516BPA 30% of wild-type laminC-Flag without amber codons. Thus, pBPA incorporation efficiency depends on the site of the amber stop codon within the laminC gene.

### UV_365nm_-dependent crosslinking

**FIGURE 2:**
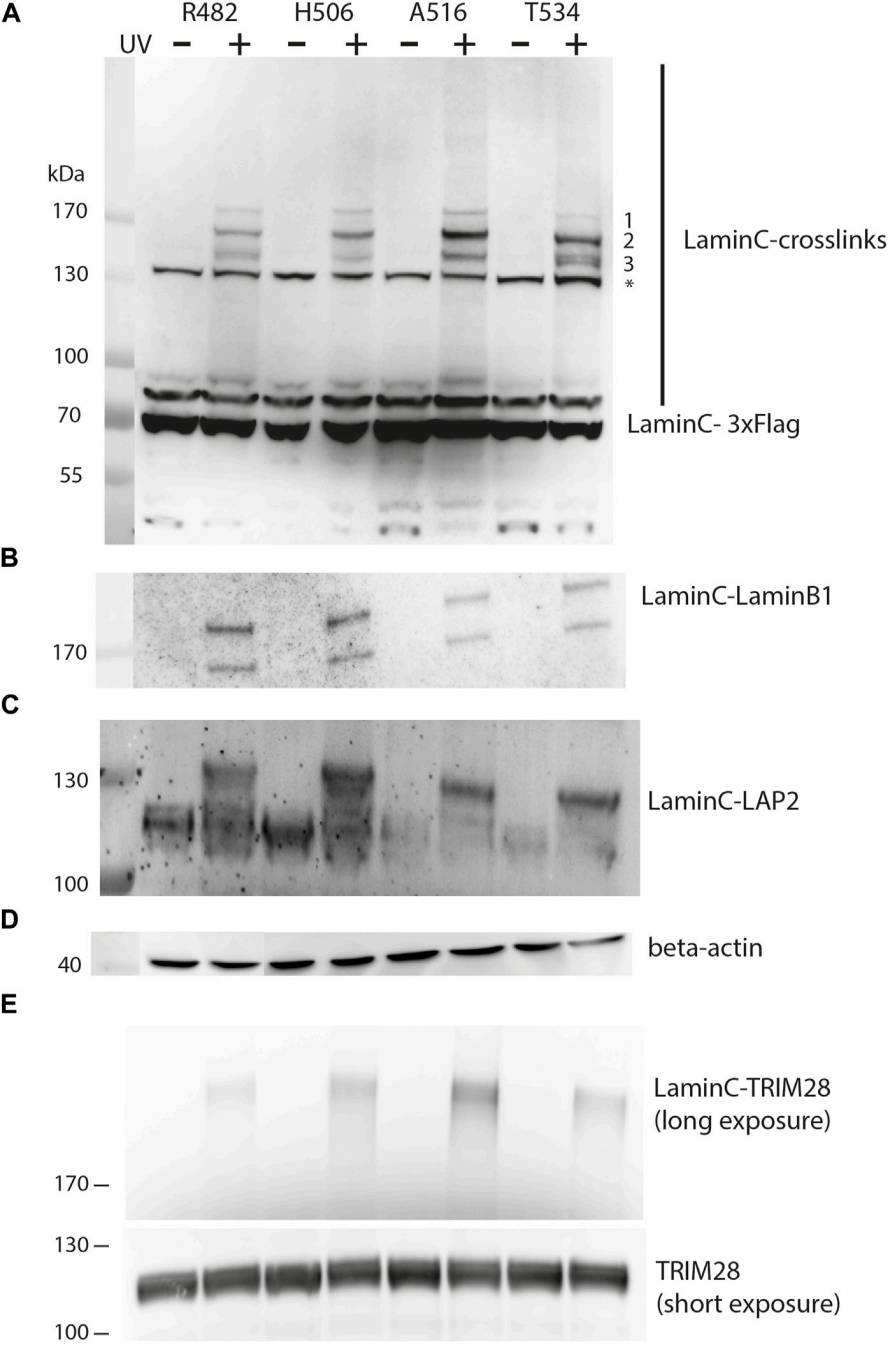
UV-dependent crosslinking pattern of individual sites within laminC. HEK293 cells were transiently transfected as described in Figure 1. All cells were cultivated in medium containing pBPA. Cells were exposed to UV_365nm_ as indicated and lysates analyzed by Western blot using **(A)** anti-Flag (major crosslink products marked with 1–3), **(B)** anti-laminB1, **(C)** anti-LAP2, **(D)** anti-β-actin (loading control) and **(E)** anti-TRIM28 antibody. The asterisk marks a non-specifically cross-reacting band of the anti-Flag antibodies. Panel **(E)** was obtained with a different set of samples, therefore regular TRIM28 is shown as a separate loading control.

Next, we expressed the laminC-3xFlag amber mutants together with the amber suppressor plasmid in HEK293 cells in the presence of pBPA, irradiated the cells with UV and analyzed the crosslink products formed by Western blot (Figure 2). The appearance of additional bands upon UV-treatment is a result of the photo-activatable pBPA crosslinker capturing interacting proteins from the corresponding site of the Ig-fold of laminC.

Prominent crosslink products could be detected between 130 and 170 kDa, while less intense crosslink products are present at higher apparent molecular weight than 170 kDa. This may reflect the decreasing transfer efficiency of large proteins in Western blots. While the crosslinking pattern of different laminC-pBPA mutants is quite similar, there are differences in the intensity of the three major bands. The slowest migrating band (>170 kDa, 1) is uniform in intensity, the second band (migrating slightly faster than 170 kDa, 2) is strongest for A516, whereas Band 3 migrates as a double band only in T534 and to some extent in R482 (Figure 2A).

From these comparisons, we conclude that pBPA traps different types of laminC interaction partners or different quantities when incorporated at different sites. These differences are not due to different pBPA incorporation efficiencies because, for example, R482 and T534 install pBPA to a similar extent but display different intensities in their crosslink pattern (Figure 1C, Figure 2A). Additionally, the band intensities of R482 and H506 are very similar, but their pBPA dependent expression is significantly different.

In order to identify the crosslink partners, we analyzed the samples with antibodies against known interactors of laminA/C. First, we tested for laminB1 because it has been shown by BioID that it is located near laminC (Xie et al., 2016). Indeed, we found the laminC-laminB1 interaction as shown in Figure 2B. The two bands on the Western blot are most likely alternative splice variants of laminB1. Secondly, we examined LAP2, an inner membrane protein which interacts with DNA-binding proteins and lamins (Dechat et al., 2000), using an antibody directed against all LAP2 isoforms. In fact, we identified laminC-LAP2 interactions for all four positions within the Ig-fold (Figure 2C). We also identified TRIM28 (KAP1), a protein which is involved in transcriptional control and gene silencing (Figure 2E) (Randolph et al., 2022). TRIM28 is recruited to promoter regions through interactions with Krüppel-associated box (KRAB) domain-containing Zinc Finger DNA-binding proteins and subsequently attracts epigenetic silencing factors such as histone deacetylases, H3 K9 methyltransferases and heterochromatin protein 1 (HP1).

We conclude that our method is very robust in eukaryotic cells to study laminC interactions. A particular strength of this method is the fact that it allows the quantification of lamin-protein interactions in living cells. Hence, we next tested whether we can detect differences in the crosslinking pattern of a laminC-pBPA mutant when exposed to different conditions.

### Phosphomimetic mutations of cdk1 sites alter crosslink pattern of laminC

A-type lamins have been shown to become mobile within the nucleus in response to mechanical stress signaling. One known mechanism for solubilizing A-type lamins is phosphorylation (Athirasala et al., 2017). This is also an important mechanism in mitosis and interphase cells. Kochin et al. investigated the influence of laminA phosphorylation sites on nuclear organization and dynamics in interphase cells by expressing phosphomimetic mutants using a plasmid-based system. The authors found that two sites, S22 and S392, targeted by Cdk1, are not only important and phosphorylated during mitosis, but also during interphase.

**FIGURE 3:**
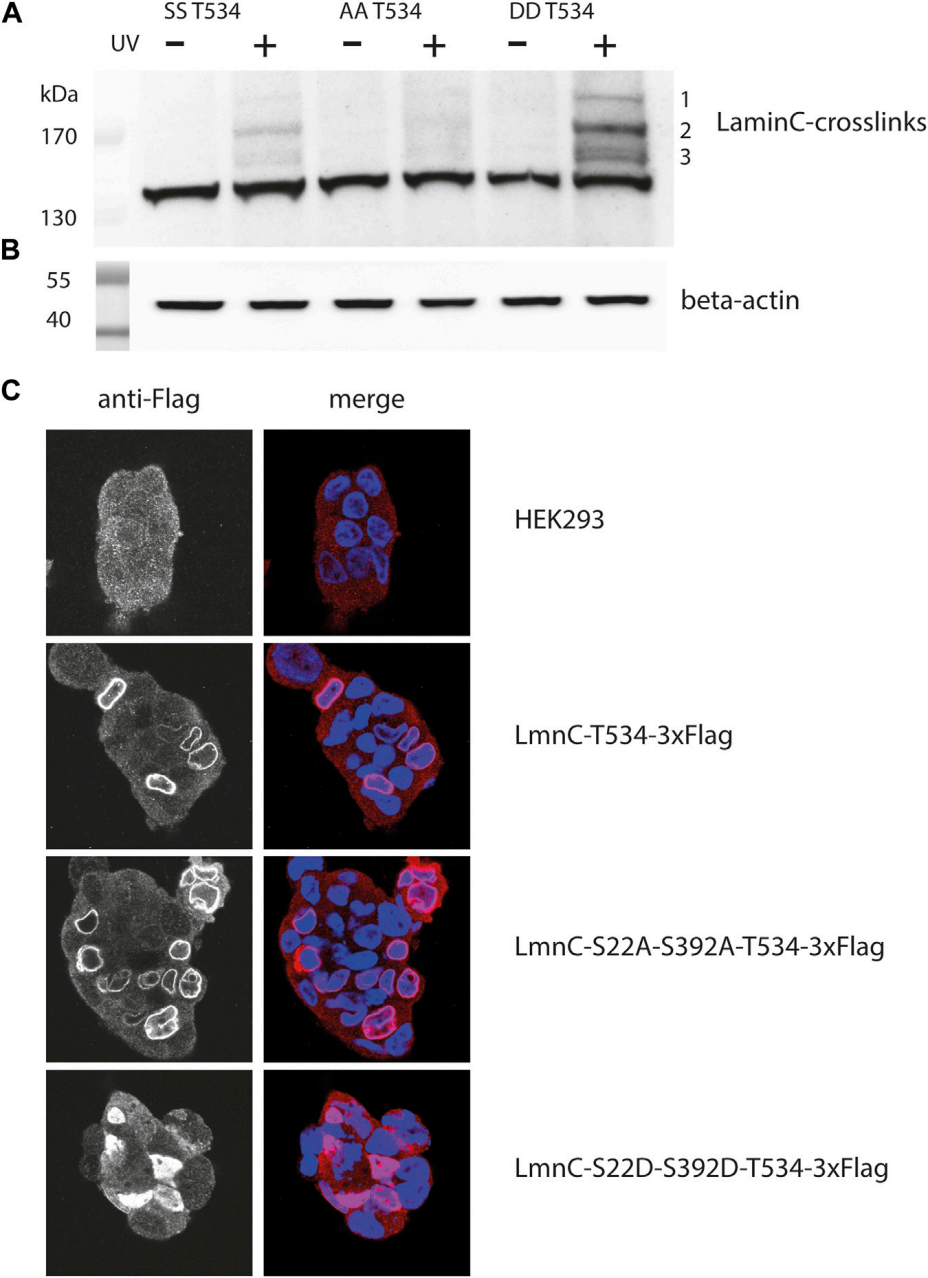
Crosslink patterns of laminC-T534pBPA phosphorylation-site mutants. Cells were co-transfected with the amber suppressor plasmid and pLmnC-T534-3x-Flag plasmids containing the indicated phosphorylation-site mutations, grown in the presence of pBPA and exposed to UV_365nm_ as indicated. Proteins were analyzed by Western blot and detected with **(A)** anti-Flag (major crosslinks are numbered with 1–3), **(B)** β-actin antibody (loading control). **(C)** Localization of the individual cdk1 mutated proteins within the nucleus; blue = DAPI stain, red = Cy3 anti-Flag stain.

Accordingly, we mutated both Cdk1 sites in laminC to either alanine or aspartate to mimic the dephosphorylated and phosphorylated states, respectively. In these plasmids, we introduced the amber stop codon at position T534. Compared to the parent laminC-T534BPA mutant (Figure 3A, labelled as “SS”), the crosslinks of the Cdk1 double alanine mutant (“AA”) are barely visible, whereas the intensity of all crosslink bands in the Cdk1 double aspartate mutant (“DD”) increases.

This was unexpected, because this phosphorylation-deficient mutant should remain associated with the lamina (Kochin et al., 2014) and therefore should display a crosslink pattern similar to the parent protein. Indeed, the laminC-T534BPA-Flag double alanine mutant localized to the lamina like the wild-type (Figure 3C). We speculate that the phosphorylation-deficient laminC mutant forms a dense, impenetrable network, that physically excludes potential interaction partners.

On the other hand, it was surprising that the crosslink pattern of the phosphomimetic mutant increased in intensity (Figure 3A) since this mutated laminC should only partially associate with the lamina (Kochin et al., 2014). Indeed, the laminC-T534-3xFlag double aspartate mutant was distributed within the entire nucleoplasm (Figure 3C). We think that a potential mobile laminC is more accessible to possible interaction partners than the mixture of phosphorylated/non-phosphorylated wild-type laminC. We obtained very similar results when we analyzed phosphorylation-site mutants of laminC H506BPA (Supplementary Figure S2).

### Crosslink pattern of synchronized cells

During mitosis, the components of the lamina experience a dramatic rearrangement: Lamins for instance become hyperphosphorylated, more soluble and mobile (Torvaldson et al., 2015). Tracking such a cellular event in a time-resolved manner with respect to the proteins involved provides valuable insights into cellular dynamics (Wilkins et al., 2014).

To obtain insights into the cell cycle-dependent dynamics of the lamin interaction network, we used our approach to investigate early prophase cells. Therefore, we blocked HEK293 cells transiently transfected with pLmnC-T534TAG-3xFlag and p*Ec*BPARS/4xtRNA_CUA_ with SLTC (S-trityl-L-cysteine, an inhibitor that blocks Eg5, a mitotic kinesin required to build and maintain a bipolar spindle) and analyzed crosslinked proteins by Western blot. Alternatively, we first synchronized the cells with thymidine in S phase and subsequently released them into a prometaphase block by nocodazole, which prevents spindle formation by interfering with microtubules.

**FIGURE 4:**
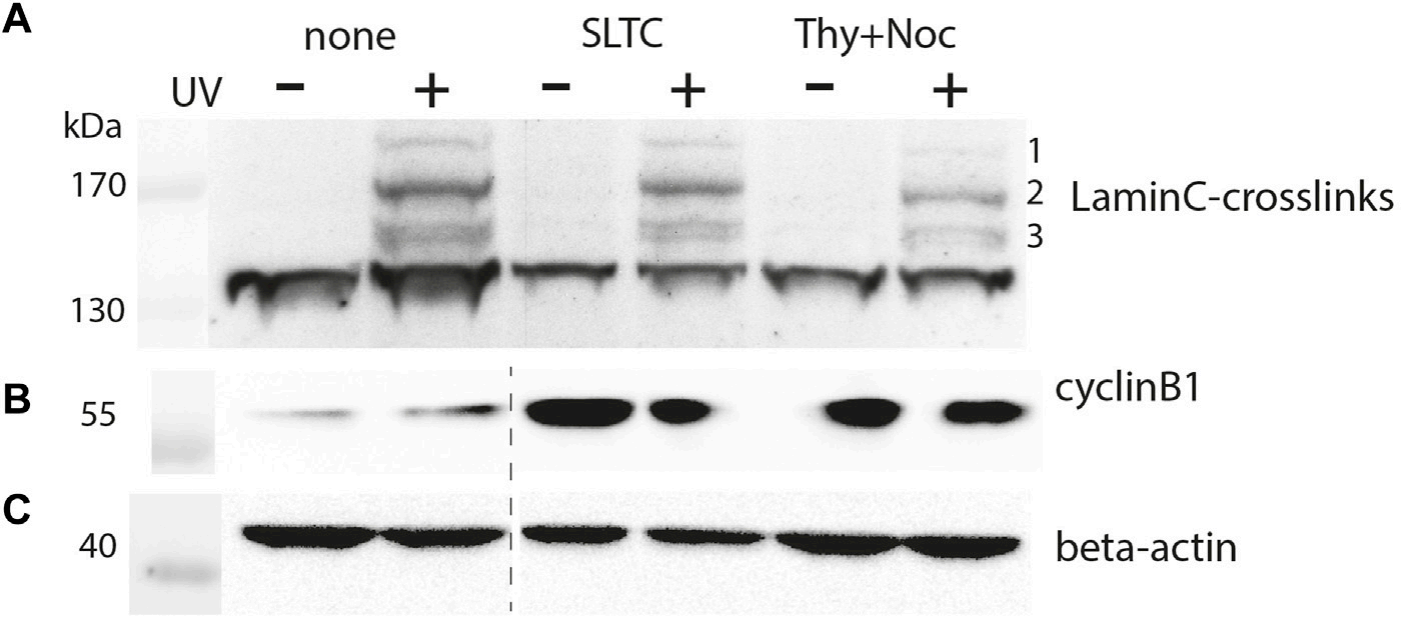
Crosslink pattern of cells synchronized in M-phase. Cells were transiently co-transfected with pLmnC-T534-3xFlag and the amber suppressor plasmid, synchronized in early M-phase as indicated and analyzed by SDS-PAGE and Western blot. Samples marked as “none” are derived from asynchronised cells, “SLTC” are samples from SLTC-inhibited cells and “Thy+Noc” marks samples from cells blocked first in S-phase and then released into nocodazole block. Western blot detection with **(A)** anti-Flag, **(B)** anti-cyclinB1 (to probe the cell cycle stage) and **(C)** anti-β-actin antibodies. The dashed line in panel **(B,C)** indicates the position of an irrelevant lane that has been cut away.

Figure 4 shows the crosslinking pattern obtained with anti-Flag IgG for the T534 site. The patterns of interphase and prophase cells are similar but the intensities of the bands are reduced for the latter. By densitometry we found the intensity of the major crosslink product (#2 in Figure 4A), normalized to the β-actin signal, to decrease to 66% in the SLTC and to 24% in the thymidine/nocodazole treated sample. This suggests that the typical interaction network of the nuclear lamina is remodeled in mitosis. Since synchronization is usually incomplete, the extent to which interactions are lost in mitosis might be underestimated by this experiment.

## Discussion

We established a method to trap interaction partners of the nuclear lamina *in vivo* that facilitates mapping and quantifying the interactions of candidate proteins in different cellular states. Microscopic analysis confirmed pBPA-dependent expression and the successful incorporation of several laminC-pBPA mutants in the nuclear lamina. Our crosslinking experiments identified interactions of laminB1 and LAP2 with laminC, which had previously been observed by BioID (Xie et al., 2016). As a new interaction partner of laminC we found TRIM28, which is particular interesting since it provides another link to the lamina`s function in gene silencing. The interaction of TRIM28 with laminA had been observed previously using CoIP combined with formaldehyde-crosslinking (Kubben et al., 2010). The interaction of TRIM28 with the nuclear lamina might contribute to the sequestering of heterochromatin at the nuclear periphery, which is generally a transcriptionally repressive environment (Gonzalez-Sandoval and Gasser, 2016).

In addition to preserving the physiological context, site-directed *in vivo* crosslinking provides additional information on the site of interaction (the Ig-fold) and allows its quantification between mutants or cell cycle stages. Phosphorylation of lamins heralds their disassembly during nuclear envelope breakdown in mitosis. Interestingly, phosphomimetic mutations in laminC result in a strong general increase in protein-lamin crosslinks without a pronounced shift in relative abundance (Figure 3), suggesting that the assembled filaments and the soluble pool of laminC are engaged in a similar set of interactions. Perhaps, the soluble pool is more easily accessible to binding proteins, leading to a higher general crosslink yield.

In comparison, blocking cells in mitosis leads to a reduction in crosslinking efficiency. This observation, which appears contradictory to the phosphomimetic mutant data at first sight (an increased level of crosslinking would be expected, because lamins are phosphorylated and the lamina is disassembled in mitosis), can be explained by considering that under these conditions the entire lamina is solubilized. Although this may increase the accessibility of the lamins, it does not lead to the formation of separate pools that would interact to different degrees. Moreover, the phosphomimetic mutants were crosslinked in unsynchronized cells, which are predominantly in interphase with an intact nucleus. Nuclear envelope breakdown in mitosis probably creates a radically different environment in which lamin interactors are diluted in the cytoplasma.

Our crosslinking approach can be extended to detect other lamin interaction partners. We followed a candidate approach using Western blot and commercially available antibodies. With the help of stable cell lines (Elsässer et al., 2016), it should be possible to scale up the crosslinking reactions for affinity purification and identification of novel lamin interaction partners by mass spectrometry. We observed little variation in the crosslinking patterns between different pBPA-incorporation sites, indicating that the dominant interactions cover a large surface on the Ig-fold. To obtain a more comprehensive picture of laminC interactions, an amber-mutant screen along the whole protein is required.

In combination with advanced microscopy and mass-spectrometry data from proximity-labelling methods, results acquired by *in vivo* site-directed crosslinking will significantly contribute to a more comprehensive understanding of the lamina network and associated processes. Our approach is ideally suited to quantify the influence of disease-related mutations on the strength of lamin interactions.

## Data Availability

The original contributions presented in the study are included in the article/Supplementary Material, further inquiries can be directed to the corresponding author.
