# Supplementary material for: A method to map the interaction network of the nuclear lamina with genetically encoded photo-crosslinkers in vivo
Source: Front Chem. 2022 Aug 30;10:905794. doi: 10.3389/fchem.2022.905794 (PMC9468544; doi:10.3389/fchem.2022.905794)
Supplement: Supplementary file 1 [file DataSheet1.PDF]

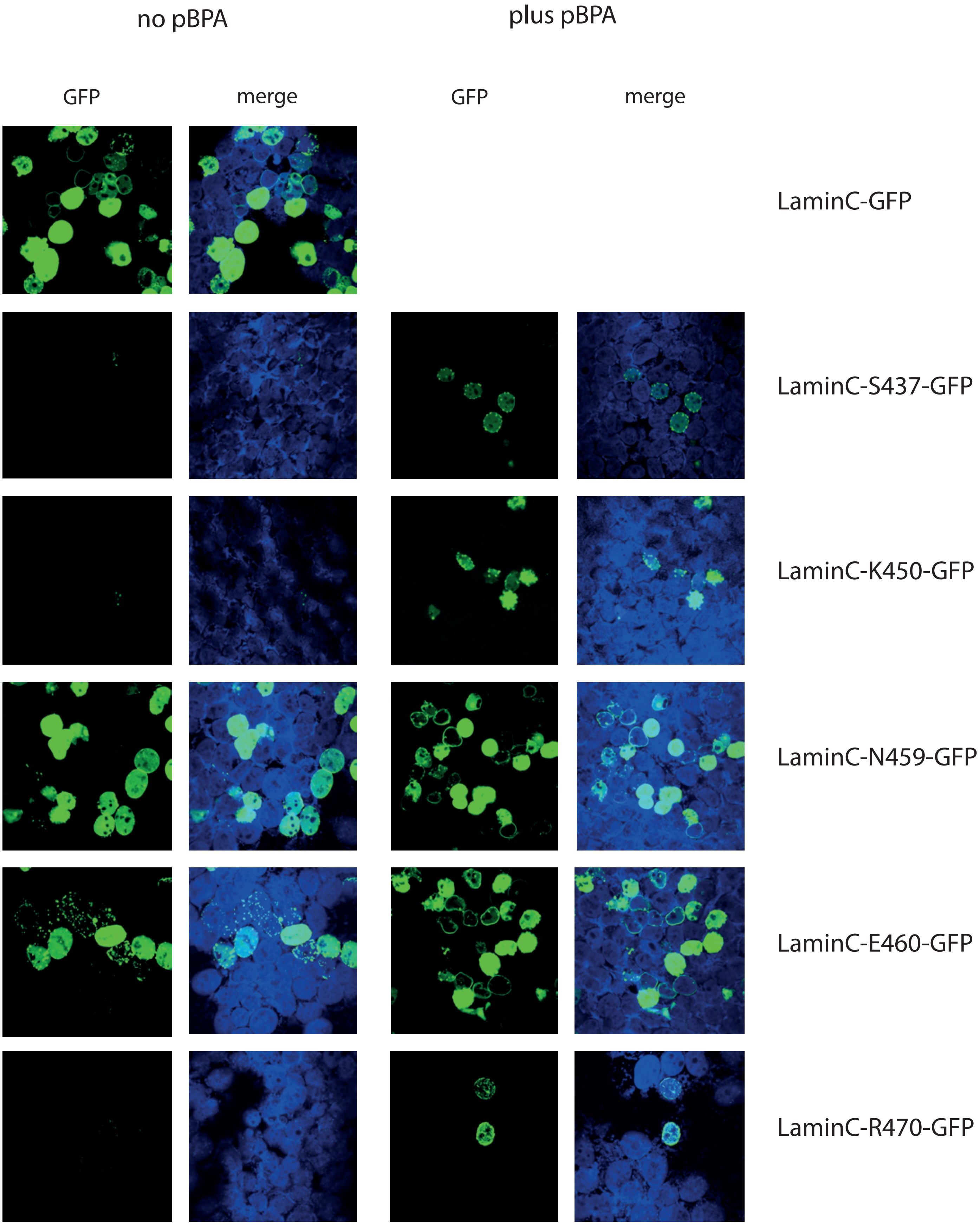

Supplementary Figure 1, part I: HEK293 cells cotransfected with a pLmnC-GFP construct with an amber mutation at the indicated site and the p4xtRNA/synthetasepBPA plasmid, incubated w/wo pBPA, and analysed by LSM. LaminC-GFP fusion proteins are shown in green, DNA is colored in blue.

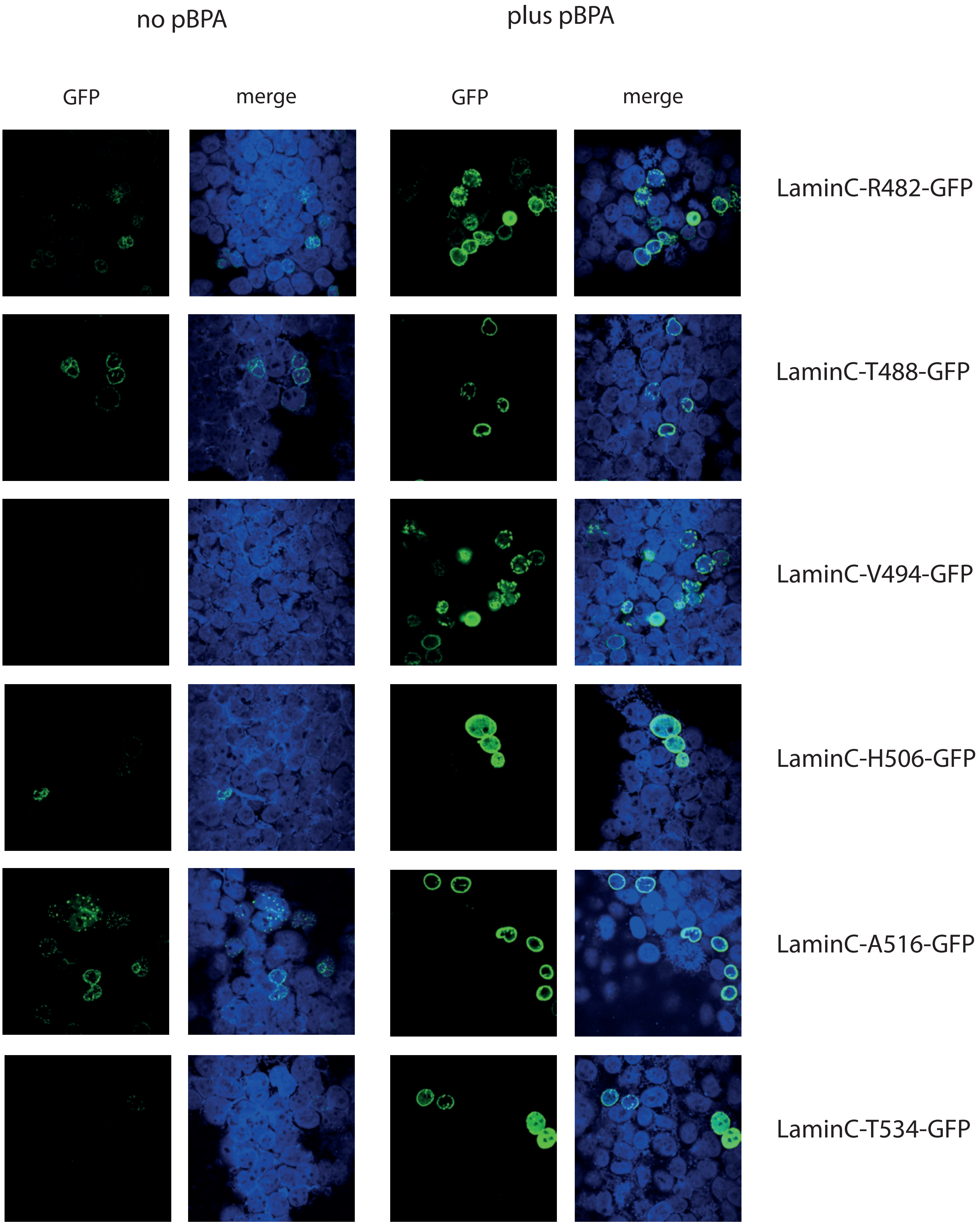

Supplementary Figure 1, part II: HEK293 cells cotransfected with a pLmnC-GFP construct with an amber mutation at the indicated site and the p4xtRNA/synthetasepBPA plasmid, incubated w/wo pBPA, and analysed by LSM. LaminC-GFP fusion proteins are shown in green, DNA is colored in blue.

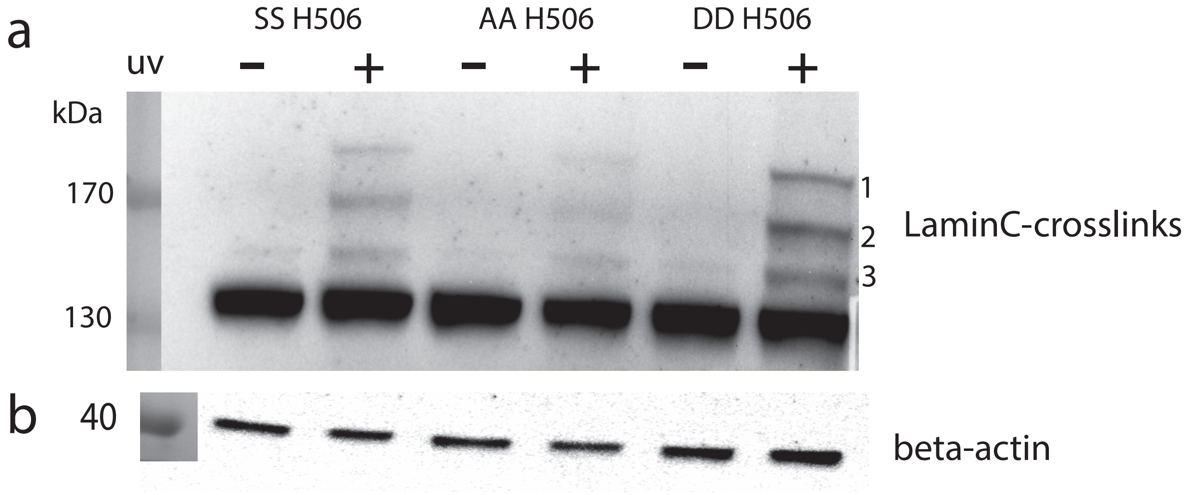

Supplementary figure 2: Crosslink pattern of cdk1 mutated sites S22 and S392 of amber mutant H506. The pLmnC-H506-3x-Flag plasmid was used in the wild type form "SS", with cdk1 sites S22 and S392 mutated to alanine "AA" or to aspartate "DD". In combination with the p4xtRNA/synthetasepBPA system these plasmids were transiently expressed. Cells were grown in pBPA containing medium and exposed to UV (365 nm) as indicated. In (a) laminC crosslink products are visualized using an anti-Flag antibody (major crosslinks are numbered with 1-3), (b) in (b) with anti-beta-actin.
